# Glutathione supplementation improves fat graft survival by inhibiting ferroptosis via the SLC7A11/GPX4 axis

**DOI:** 10.1186/s13287-024-03644-0

**Published:** 2024-01-30

**Authors:** Zehua Li, Jinqiang Lu, Zhiqin Dong, Jiaji Liang, Shenghong Li, Wenwen Han, Taixing Cui, Hongwei Liu

**Affiliations:** 1 Department of Plastic Surgery, The First Affiliated Hospital of Jinan University, Guangzhou, Guangdong People’s Republic of China; 2 Key Laboratory of Regenerative Medicine, Ministry of Education, Guangzhou, Guangdong People’s Republic of China; 3 Department of Medical Pharmacology and Physiology, Dalton Cardiovascular Research Center, School of Medicine, University of Missouri, Columbia, MO 65211 USA

**Keywords:** Adipose-derived stem cell, Fat graft, Ferroptosis, Glutathione, Lipotransfer

## Abstract

**Background:**

Autologous fat grafting is hampered by unpredictable graft survival, which is potentially regulated by ferroptosis. Glutathione (GSH), a powerful antioxidant used in tissue preservation, has ferroptosis-regulating activity; however, its effects on fat grafts are unclear. This study investigated the effects and mechanisms of GSH in fat graft survival.

**Methods:**

Human lipoaspirates were transplanted subcutaneously into the backs of normal saline-treated (control) or GSH-treated nude mice. Graft survival was evaluated by magnetic resonance imaging and histology. RNA sequencing was performed to identify differentially expressed genes and enriched pathways. GSH activity was evaluated in vitro using an oxygen and glucose deprivation (OGD) model of adipose-derived stem cells.

**Results:**

Compared with control group, GSH induced better outcomes, including superior graft retention, appearance, and histological structures. RNA sequencing suggested enhanced negative regulation of ferroptosis in the GSH-treated grafts, which showed reduced lipid peroxides, better mitochondrial ultrastructure, and SLC7A11/GPX4 axis activation. In vitro, OGD-induced ferroptosis was ameliorated by GSH, which restored cell proliferation, reduced oxidative stress, and upregulated ferroptosis defense factors.

**Conclusions:**

Our study confirms that ferroptosis participates in regulating fat graft survival and that GSH exerts a protective effect by inhibiting ferroptosis. GSH-assisted lipotransfer is a promising therapeutic strategy for future clinical application.

**Supplementary Information:**

The online version contains supplementary material available at 10.1186/s13287-024-03644-0.

## Background

Fat transplantation is an important cosmetic and reconstructive technique in plastic surgery for wound healing, soft-tissue defect repairing, and body shaping. However, its application is limited by the unpredictability of graft survival. Poor graft survival is mainly presented as an absorption rate of up to 80%, liquefaction necrosis, and fiber calcification, which ultimately lead to unsatisfactory or failed surgical results [1]. Many strategies have been proposed to promote fat graft survival, but few are clinically effective. Therefore, the use of exogenous biologics, combined with advanced clinical methodologies and surgical techniques, represents a promising solution to safely promoting graft transplant success [2].

Glutathione (GSH) is used in the preservation of multiple cell and tissue types because of its role in maintaining intracellular stability and defense mechanisms. Changes in its activity are associated with the activation of redox signaling, which in turn regulates the cell death machinery [3]. Previous studies have shown that intravenous GSH administration was effective in preventing renal injury resulting from coronary angiography [4]; early and prolonged application of a GSH infusion improved cardiac repair and prevented infarct expansion in patients with myocardial infarction undergoing primary percutaneous coronary intervention [5]; oral GSH administration sensitized healthy and diabetic individuals to insulin [6]; and the perioperative use of GSH alleviated postoperative inflammation and promoted recovery [7]. However, the effects of GSH on fat grafts are unclear. Fat grafts commonly undergo ischemia and inflammation after transplantation, and most cells die within one week with only the ~ 300-μm superficial layer surviving [8]. Notably, N-acetylcysteine, a precursor of GSH, that has been reported to be able to improve graft survival [9, 10]. Therefore, we have reason to believe that GSH could potentially regulate the survival of fat cells after transplantation.

Cell death is an important determinant of graft survival [11], and research on the related mechanisms has increased in recent years [12]. Although various studies have emphasized the importance of necrosis and apoptosis, the current paradigm acknowledges various cell death pathways. Ewa [13] and Kim [14] reported that coenzyme Q10 (CoQ10) and deferoxamine (DFX) promoted fat graft survival, respectively. While CoQ10 is an antioxidant and DFX is an iron chelator, both exert anti-ferroptotic effects. Although not explicitly indicated in these studies, we speculate that these interventions exerted their protective effects on fat grafts by inhibiting ferroptosis. Several studies have shown that GSH has a dual regulatory effect on ferroptosis [15, 16]. GSH depletion can trigger ferroptosis, which has been exploited in cancer treatments [17], and supplementation with GSH is utilized by glutathione peroxidase 4 (GPX4) to prevent lipid peroxidation, in turn inhibiting ferroptosis. The GSH-mediated regulation of ferroptosis has shown some promising results in the treatment of ischemic injury to the liver, kidney, brain, and heart as well as some neurodegenerative diseases [18]. However, whether ferroptosis participates in the regulation of fat graft survival requires further research.

This study investigated the effects of GSH on fat graft survival and determined the underlying mechanism in a nude mouse model. Based on previous findings regarding the activities of GSH, we hypothesized that GSH would promote fat graft survival by suppressing ferroptosis.

## Materials and methods

### Fat grafts and animal model

**Fig. 1:**
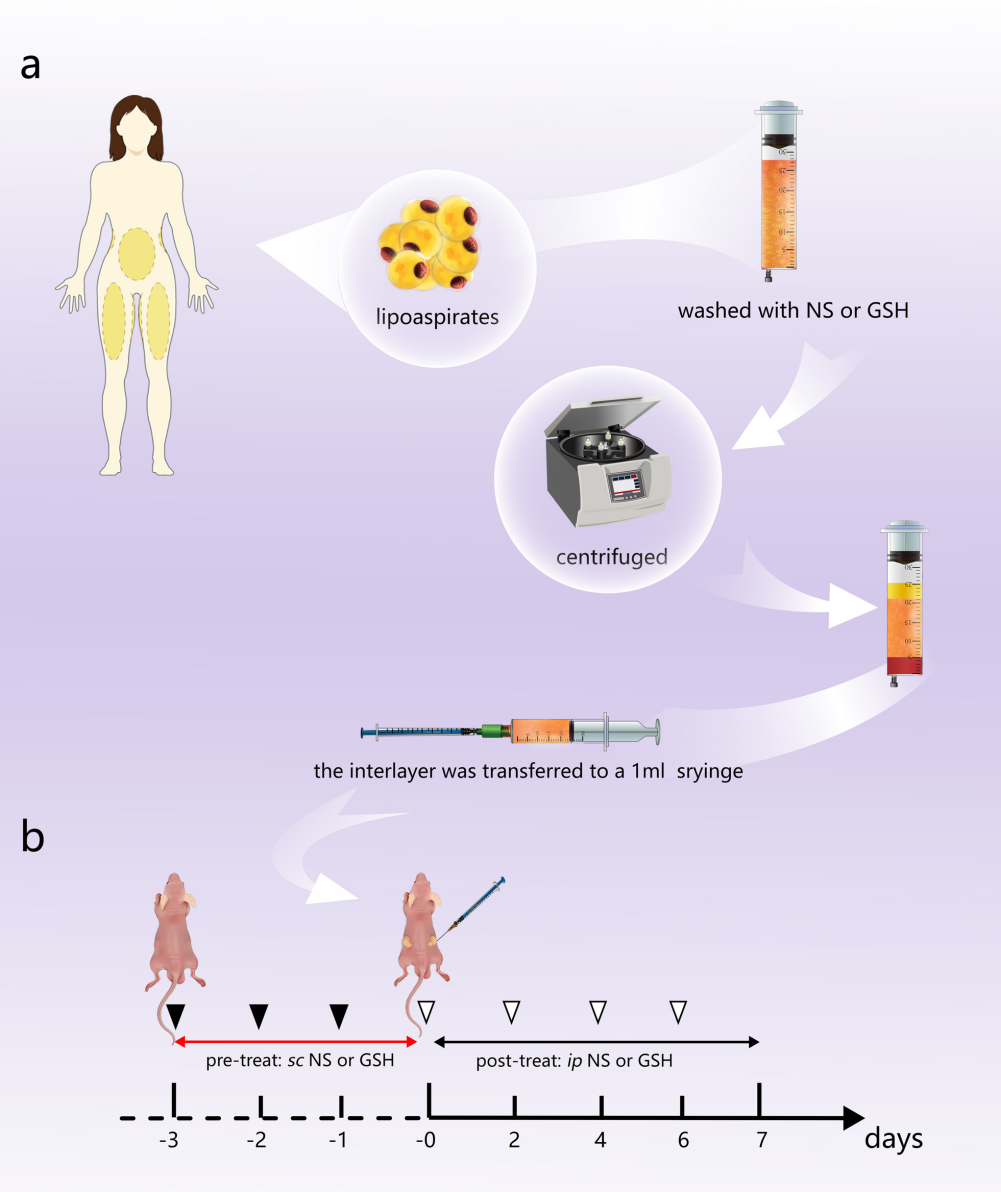
Schematic diagram of the study design. **a** Preparation of fat grafts. **b** Construction of the nude mouse model and timeline of normal saline (NS) and glutathione (GSH) treatment interventions. Black arrowheads indicate prelipotransfer subcutaneous injections; white arrowheads refer to postlipotransfer intraperitoneal injections

Human lipoaspirates were harvested from the lower abdomen and anterior thighs of five healthy women with no contraindications; informed consent was obtained prior to surgery. The lipoaspirates were washed with normal saline (NS) as a control or 1% GSH solution as an intervention, followed by centrifugation at 500 rpm for 3 min. The interlayer, representing the fat graft, was then transferred to a 1-mL syringe (Fig. 1a).

Male BALB/c-nu nude mice aged 4 weeks and weighing 18 ~ 22 g were purchased from the Experimental Animal Centre of Southern Medical University (Guangzhou, China) and housed in a specific pathogen-free air-conditioned room (25 ± 2 ℃ and 65% ± 5% humidity) with a 12-h light/dark cycle. They were randomly assigned to GSH and control groups (18 mice per group) and all surgical procedures were performed under general anesthesia with intraperitoneal injection of 1% sodium pentobarbital solution (50 mg/kg body weight). The mice in the GSH group received a subcutaneous injection of 0.5 mL 1 g/kg GSH on each side of the lower back for 3 consecutive days (-3, -2, and -1 days, prior to the start of the experiment), after which 0.2 mL of the prepared lipoaspirate was transplanted subcutaneously at the same sites of injection at day 0 of the experiment under anesthesia (Fig. 1b). On days 0, 2, 4, and 6 of the experiment, the mice received intraperitoneal injections of 0.2 mL 1 g/kg GSH and were monitored over a 3-month period. NS was used instead of GSH in the control group.

## Magnetic resonance imaging (MRI) of mice

During the early monitoring period, mice were anesthetized with 2% isoflurane through a nose cone and the survival of fat grafts was evaluated using a 9.4-T small animal MRI scanner (BioSpec 94/30, Bruker, Billerica, MA, USA), while continuously monitoring the body temperature and respiratory rate of the mice. T2-weighted scans were performed, and the images were observed using RadiAnt DICOM Viewer software (Medixant, Poznań, Poland).

## General analysis

The implanted fat grafts were harvested and photographed. The graft weights were recorded, and their volumes were measured using the liquid overflow method [19]. Both weight and volume retention rates were calculated. Subsequent to the data acquisition, the murine subjects were humanely euthanized via the administration of a lethal intraperitoneal injection of sodium pentobarbital, culminating in cervical dislocation.

## Histological analysis

Graft samples were harvested at 12 weeks after transplantation and fixed in paraformaldehyde. After serial dehydration and vitrification, the tissues were cut into 5-mm sections and subjected to hematoxylin and eosin (H&E) staining. The slides were scored using the Shoshani method to evaluate graft parameters, including the presence of intact and nucleated fat cells, cysts and vacuoles, inflammation, and fibrosis [20].

For immunofluorescence staining, sections were incubated with primary antibodies targeting perilipin (1:500; cat. no. ab3626, Abcam, Cambridge, UK), von Willebrand factor (VWF, 1:400; cat. no. GB11020, Servicebio, Wuhan, China), GPX4 (1:200; cat. no. ab125066, Abcam), and SLC7A11 (1:200; cat. no. Ab307601, Abcam), followed by incubation with secondary antibodies. Nuclei were stained with 4′,6-diamidino-2-phenylindole (cat. no. GB1012, Servicebio). The levels of positive staining were quantified as the mean fluorescence intensity using ImageJ software [21].

## RNA sequencing (RNA-seq)

RNA samples from the control and GSH groups were sequenced by Oebiotech Co. Ltd. (Shanghai, China) using an Illumina HiSeq X Ten platform to generate 150-bp paired-end reads. The raw data (fastq format) were processed using Trimmomatic to remove low-quality reads and retain clean reads. The clean reads were mapped to the human genome (GRCh38) using HISAT2. Fragments per kilobase of exon per million reads mapped were calculated for each gene using Cufflinks, and read counts for each gene were obtained using htseq-count. Differential expression analysis was performed using the DESeq R package, with *p* < 0.05 and fold change > 1.5 or < 0.667 indicating statistical significance. We performed a hierarchical cluster analysis of differentially expressed genes (DEGs) to evaluate gene expression patterns between groups and samples. Kyoto Encyclopedia of Genes and Genomes (KEGG) pathway enrichment analysis of DEGs was performed using R based on the hypergeometric distribution.

## qRT-PCR assay

Total RNA was extracted using a total RNA extraction kit (cat. No. R1200, Solarbio, Beijing, China) and reverse transcribed into cDNA using a PrimeScript RT reagent kit (cat. No. RR014B, Takara Bio, Kusatsu, Japan). qRT-PCR was carried out using SYBR Green Master Mix (cat. No. 11201ES03, Yeasen, Shanghai, China) on a CFX384 real-time PCR system (Bio-Rad Laboratories, Hercules, CA, USA). Relative mRNA expression was quantified using the 2 − ΔΔCt method. The primers are listed in Additional file 1.

## Ultrastructural analysis

Graft samples were fixed in glutaraldehyde, dehydrated, impregnated, embedded in epoxy resin and acrylic, and cut into 60-nm sections using an ultramicrotome. The morphology of mitochondria was studied by transmission electron microscopy (TEM) using a Hitachi TEM system (HT7800, Tokyo, Japan).

## Cell culture

For primary culture of adipose-derived stem cells (ADSCs), the lipoaspirates were washed with phosphate-buffered saline (PBS) and rested for 10 min at room temperature. The sedimented liquid layer containing tumescent fluid and blood components was discarded. The remaining fat particles were digested with 0.2% collagenase (Gibco, Billings, MT, USA) in a water bath shaker at 37 °C for 30 min, and the mixture was centrifuged at 1000 rpm for 5 min to remove undigested fat. After filtration and centrifugation, the cell pellet was resuspended with low glucose Dulbecco’s modified Eagle’s medium (DMEM, Gibco) containing 10% fetal bovine serum (FBS, Gibco) and 1% penicillin–streptomycin (Gibco) at 37 °C and 5% CO2 in 10-cm dishes. Cells at passages 3–5 were used for subsequent experiments.

## ADSCs identification

Flow cytometry analysis of specific mesenchymal surface markers was performed with Human MSC Analysis Kit (cat. no. 562245, BD Bioscience, USA), which contains positive markers of CD73 APC, CD90 FITC, CD105 PerCP and negative cocktail (CD11b, CD19, CD34, CD45, HLA-DR PE). ADSCs at passage 3–4 were and resuspended and incubated with the antibodies or isotype. After washing and resuspension, the sample tubes were analyzed by flow cytometry by BD FACSymphony™.

ADSCs at passage 3–4 were seeded on 12 well plate until the confluency reached about 80%. The medium was replaced with adipogenic medium in hMACs Adipogenesis Differentiation Kit (cat. no. PWL081, meilunbio, China) and osteogenic medium in hMSCs Osteogenic Differentiation Kit (cat. no. PWL080, meilunbio, China). With a 21-day culture, the cells were fixed with 4% formaldehyde and stained with Oil Red O or Alizarin Red.

## Oxygen and glucose deprivation (OGD) model and cell viability

To imitate the in vivo environment of ischemia and hypoxia after fat transplantation, ADSCs were cultured under hypoxic and glucose-free conditions to induce an OGD model. After the cells reached ~ 80% confluence under normal culture conditions, the medium was changed to glucose-free DMEM (Gibco) and the dish was placed in an anaerobic chamber (5% CO2, 95% N2) to induce OGD-related cell injury. ADSCs cultured under normal conditions were used as controls.

Cell viability was assessed using a Cell Counting Kit-8 (Dojindo, Kumamoto, Japan) according to the manufacturer’s instructions, with absorbance measured at 450 nm. The most appropriate OGD duration and GSH concentration were determined by cell viability comparisons.

The level of OGD-induced ferroptosis in ADSCs was assessed according to the viability of cells treated with the ferroptosis inhibitor Ferrostatin-1 (Fer-1) 5 μM, autophagy inhibitor 3-Methyladenine (3-MA) 5 mM, apoptosis inhibitor Z-VAD-FMK 10 μM, necroptosis inhibitor Necrostatin-1 (Nec-1) 50 μM, or ferroptosis activator erastin.

## Malondialdehyde (MDA) and superoxide dismutase (SOD) detection

The levels of MDA and SOD in graft samples were determined using a lipid peroxidation MDA assay kit (cat. no. S0131S, Beyotime, Shanghai, China) and total SOD assay kit (cat. no. S0101S, Beyotime), respectively, according to the manufacturer’s instructions. Both MDA and SOD levels were normalized per milligram of total protein.

## Determination of intracellular reactive oxygen species (ROS) levels

Total intracellular ROS levels were determined using a ROS assay kit (cat. no. S0033S, Beyotime) according to the manufacturer’s instructions. Cells treated with test compounds were washed with PBS and incubated with 10 μM 2′,7′-dichlorofluorescein diacetate (DCFH-DA) at 37 °C for 30 min, followed by washing with PBS, digestion with trypsin, and collection. The final cell suspensions were analyzed by flow cytometry to test the intensity of DCFH-DA fluorescence at excitation and emission wavelengths of 480 nm and 530 nm, respectively.

## Lipid peroxidation staining

Intracellular lipid peroxide (LPO) levels were measured using a lipid peroxidation kit (Thermo Fisher Scientific, Waltham, MA, USA) according to the manufacturer’s instructions. Adherent cultured cells in each intervention group were stained with a BODIPY 581/591 C11 probe for 30 min at 37 °C. Upon lipid peroxidation, the probe causes a fluorescence shift from red (590 nm) to green (510 nm). After being washed three times with PBS, the cells were observed and imaged under a fluorescence microscope (Nikon, Japan).

## Western blotting

Samples were lysed in RIPA buffer containing a protease inhibitor, and the total protein content was quantified using a bicinchoninic acid protein assay kit (Beyotime). Equal amounts of protein were separated by 10% sodium dodecyl sulfate–polyacrylamide gel electrophoresis and transferred onto polyvinylidene difluoride membranes. After blocking, membranes were incubated with primary antibodies against SLC7a11 (1:1000, Abcam), GPX4 (1:1,000, Abcam), and PPAR (1:1,000; cat. no. ab178860, Abcam) overnight at 4 °C followed by incubation with a horseradish peroxidase-linked secondary antibody. Blots were detected using an enhanced chemiluminescence reagent [22]. The relative protein levels were normalized to that of β-actin and quantified using ImageJ.

## Statistical analysis

Data are expressed as the mean ± standard deviation (SD) of at least three independent experiments. All analyses were performed using GraphPad Prism software (GraphPad Software, San Diego, CA, USA). Differences between groups were analyzed using a Student’s t test (two-group comparisons) or one-way analysis of variance (multiple-group comparisons) with Tukey’s post-hoc multiple comparisons test. Pearson’s correlation coefficient was calculated to assess the strength of association between qRT-PCR and RNA-sequencing data. Statistical significance was set at *p* < 0.05.

## Results

### GSH effectively improves fat graft survival

**Fig. 2:**
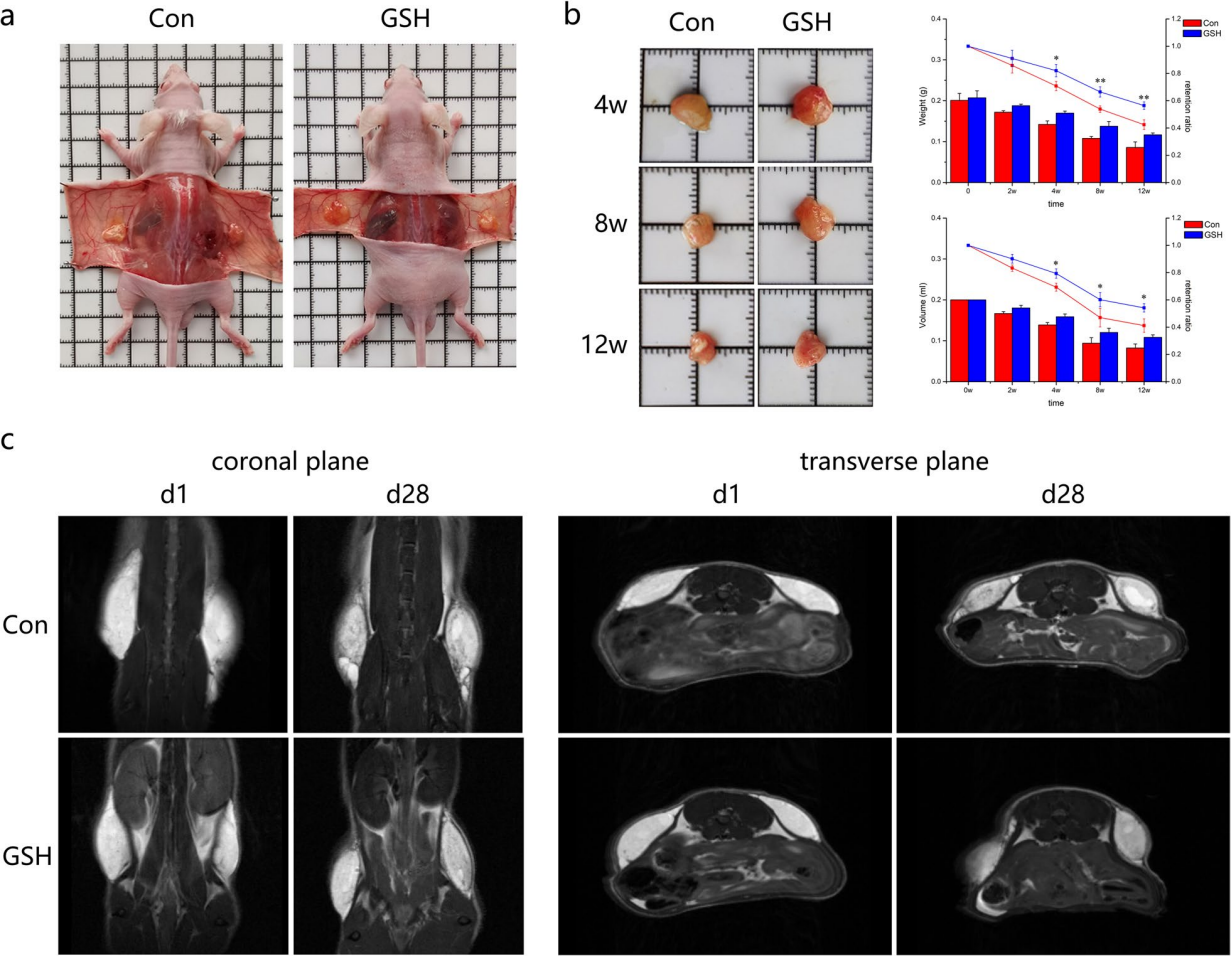
Survival of fat grafts in GSH-treated and control (Con) nude mice. **a** Survival of fat grafts in situ (n = 3). **b** Appearance of harvested fat grafts at 4, 8, and 12 weeks (left); variation in graft weights, volumes, and retention rates over time (right; n = 3). **c** Magnetic resonance imaging (MRI) imaging of implanted fat grafts at days 1 and 28. Representative T2 images of coronal and transverse planes presenting graft survival status (n = 3). Data are presented as the mean ± SD. *p < 0.05, **p < 0.01

All experimental animals survived well. The grafts in both the GSH and control groups were remodeled into a fat mass with a vascularized capsule; however, those in the GSH group were denser and larger (Fig. 2a). The volumes and weights of the grafts in both groups decreased over time. In the first 2 weeks, no differences were observed between the groups. At 4 weeks, the weight, volume, and retention rate of the grafts were lower in the control group than those in the treatment group. According to the retention curves, the shrinking of the grafts was particularly pronounced at 4–8 weeks. At the end of the experiment, the retention rate in the GSH group was superior to that in the control group (weight: 117 mg vs. 86 mg; retention ratio: 56.4% vs. 42.2%; volume: 108 μL vs. 82 μL; ratio: 54.2% vs. 41.2%; *p* < 0.05; Fig. 2b).

T2-weighted imaging of the early stage implants showed a unique and bright signal that was clearly contrasted with the surrounding muscles. Coronal and transverse images showed that the fat grafts in both groups had a homogeneous intensity on the first day after transplantation. Meanwhile, on day 28, the control-group grafts showed a well-defined lobulated mass of inhomogeneous signal intensity with a hypointense rim, corresponding to a low survival status. In addition, local necrosis was evident from the centralized globular hyperintense signal in both coronal and transverse images. In contrast, T2-weighted imaging of the GSH-treated grafts showed a homogeneous signal intensity, which indicated good survival. These preliminary results suggested that GSH can improve the survival of fat grafts (Fig. 2c).

## GSH-treated fat grafts present a better histological structure

**Fig. 3:**
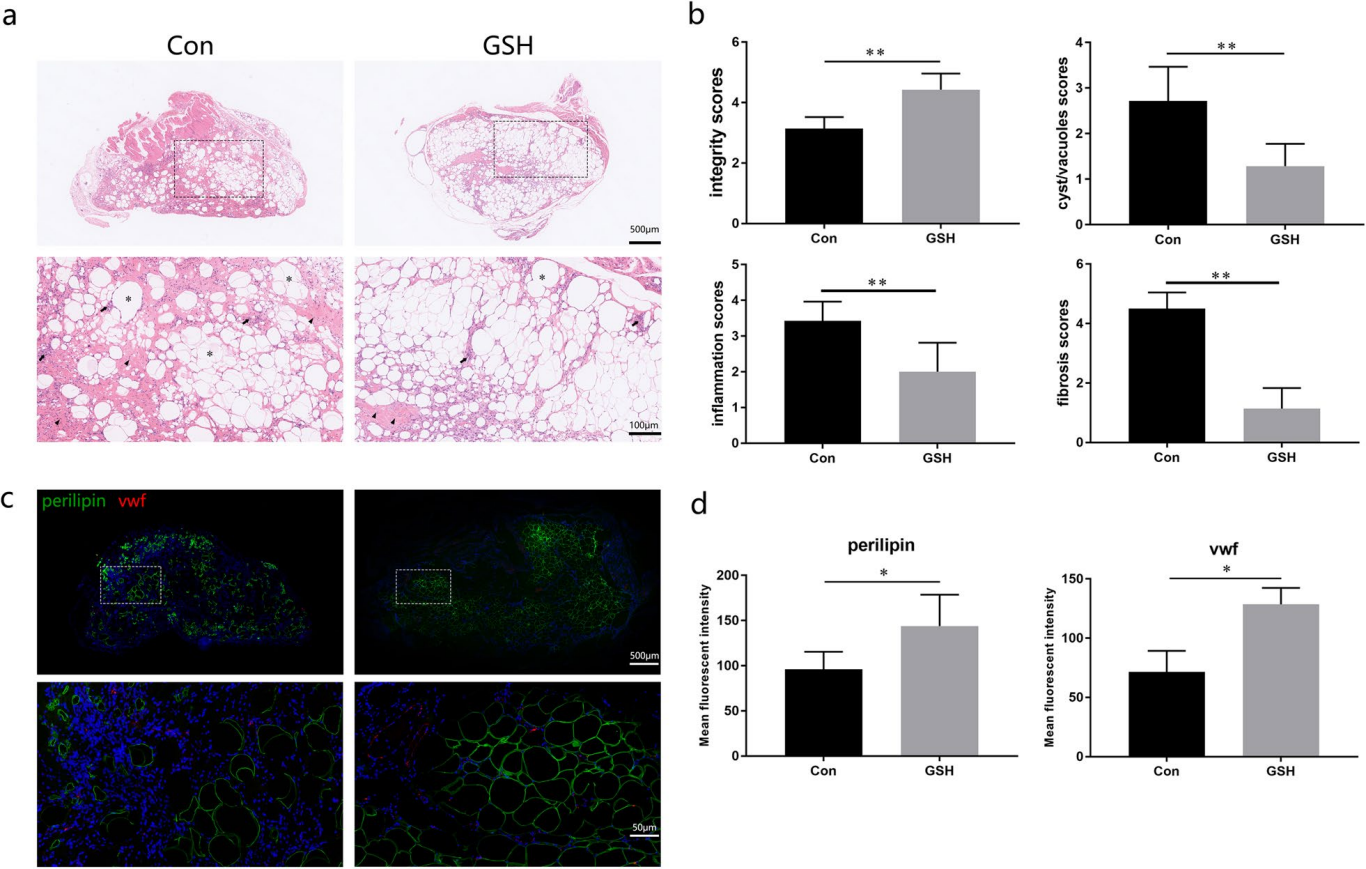
Histological analysis of fat grafts in the GSH-treated and control (Con) groups. **a** Representative images of H&E-stained fat grafts at week 12. **b** H&E scoring of cell integrity, tissue inflammation, vacuolation, and fibrosis (n = 7). Asterisk, vacuoles. Triangle, fibrotic cells. Arrow, inflammatory cells **c** Immunofluorescence staining for perilipin (green) and von Willebrand factor (vWF, red) in fat grafts between treatment groups. The bottom images are the magnified fields contained in the above dotted frames **d** Quantification of positive perilipin and vWF staining based on mean fluorescence intensity (n = 3). Data are presented as the mean ± SD. *p < 0.05, **p < 0.01

H&E staining showed that the fat grafts in the GSH-treated group exhibited better morphologic integrity, with fewer vacuoles, fibrotic cells, and inflammatory cells (Fig. 3a, b). We evaluated adipogenic and angiogenic activities by immunofluorescence and observed a higher number of intact perilipin-positive mature adipocytes in the GSH-treated group than in the control group. Positive vWF staining in the GSH-treated group confirmed the occurrence of vascular lumens, whereas the scattered vWF fluorescence in the control group demonstrated limited angiogenesis (Fig. 3c, d).

## Ferroptosis-inhibiting genes are enriched by GSH treatment

**Fig. 4:**
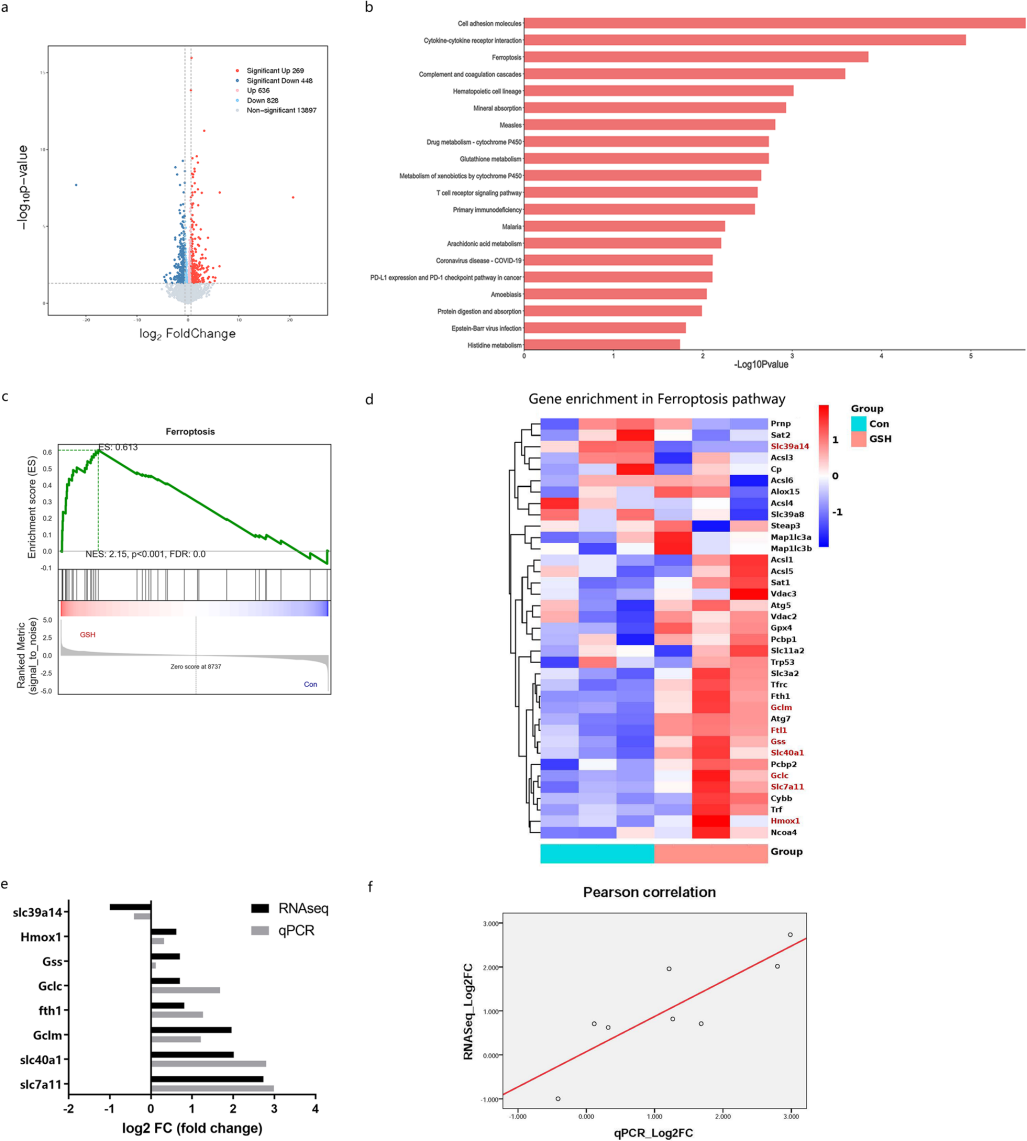
Transcriptomic analysis of fat grafts from the GSH-treated and control (Con) groups. **a** Volcano plot of DEGs between the control and GSH groups (*p* < 0.05, fold change > 1.5 or < 0.667). **b** KEGG pathway enrichment analysis of DEGs. Ferroptosis was ranked third among the top-20 enriched pathways. **c** Gene set enrichment analysis of ferroptosis pathways. **d** Heatmap of genes involved in ferroptosis. Significant DEGs are shown in red. **e** Validation of DEGs by qRT-PCR. **f** Pearson’s correlation analysis of DEG fold-change levels determined by RNA-seq and qRT-PCR. Solid line indicates the fitting curve

We used RNA sequencing (RNA-seq) to identify differentially expressed genes (DEGs) between the treatment groups (Fig. 4a) and found that ferroptosis was among the top 20 enriched Kyoto Encyclopedia of Genes and Genomes (KEGG) pathways (Fig. 4b). Relative to “cell growth and death” and “aging,” ferroptosis had the highest enrichment score (Additional file 2: Figure S1). Additionally, gene set enrichment analysis confirmed that ferroptosis was positively related to GSH treatment (Fig. 4c). We identified eight DEGs in the ferroptosis signaling pathway (Fig. 4d), among which six ferroptosis-inhibiting genes (Slc7a11, Slc40a1, Gclm, Fth1, Gclc, and Gss) were upregulated, one ferroptosis-promoting gene (Slc39a14) was downregulated, and one ferroptosis-promoting gene (Hmox1) was upregulated. Collectively, these results support our hypothesis that GSH promoted fat graft survival by inhibiting ferroptosis. The transcriptomic results were verified by quantitative reverse transcription-polymerase chain reaction (qRT-PCR) analysis (Fig. 4e). The RNA-seq and qRT-PCR results for both up- and downregulated genes showed a strong positive association (r = 0.854, *p* = 0.007; Fig. 4f).

## GSH protects transplanted fat grafts against ferroptosis

**Fig. 5:**
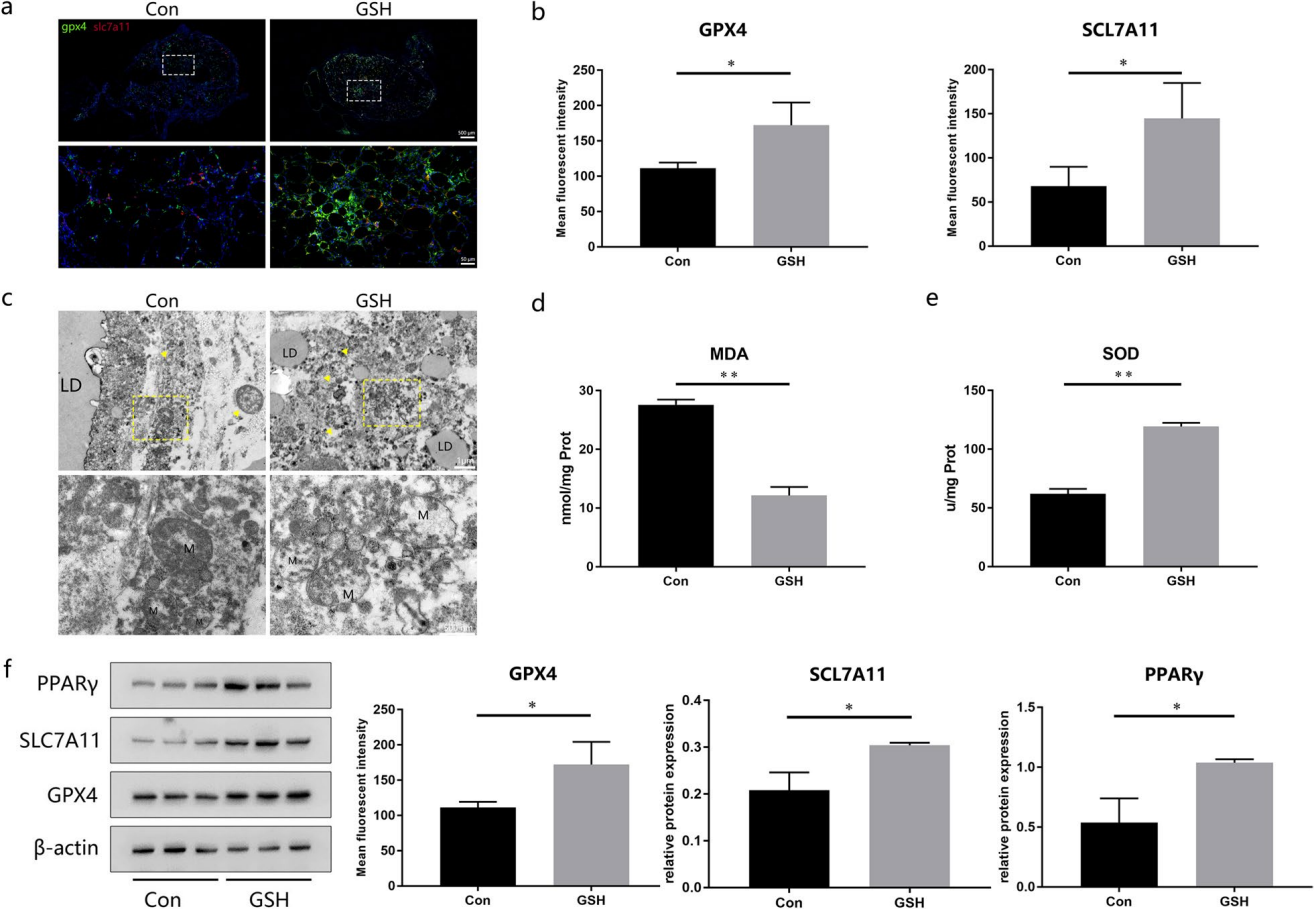
Effects of GSH supplementation on ferroptosis in fat grafts. **a** Immunofluorescence staining for GPX4 (green) and SLC7A11 (red) in fat grafts from control and GSH-treated mice. The bottom images are the magnified fields contained in the above dotted frames. **b** Mean fluorescence intensity of positive GPX4 and SLC7A11 staining (n = 3). **c** Ultrastructure of fat grafts based on TEM. The bottom images are the magnified fields contained in the above dotted frames (n = 3). Arrowheads indicate mitochondria. M: mitochondrion; LD: lipid drop. **d**, **e** Malondialdehyde (MDA) and superoxide dismutase (SOD) levels in control and GSH-treated grafts (n = 3). **f** Protein expression and quantification of GPX4, SLC7A11, and PPARγ in fat grafts (n = 3). Full-length blots are presented in Additional file 4. Data are presented as the mean ± SD. *p < 0.05, **p < 0.01

We examined the levels of ferroptosis in the transplanted fat grafts by immunofluorescence and observed a stronger positive staining for the anti-ferroptosis markers GPX4 and SLC7A11 in the GSH-treated group than in the control group (Fig. 5a, b). Mitochondria in the control group consistently showed a decrease in size and an increase in membrane density according to TEM imaging. In contrast, the GSH-treated samples appeared relatively normal, and their mitochondria were characterized by abundant, large, and clear cristae (Fig. 5c). The levels of the oxidative stress indicators MDA and SOD suggested an enhanced oxidation state in the control group, while the opposite trends were observed in the GSH-treated group (Fig. 5d, e). Western blotting showed that both SLC7A11 and GPX4, which activate inhibitory signals, were expressed at higher levels in the GSH-treated group than in the control group, similar to the expression pattern of the adipogenesis-related marker PPARγ (Fig. 5f). Collectively, these findings further confirmed our hypothesis that GSH exerts a protective effect on fat grafts by inhibiting ferroptosis.

## OGD triggers ferroptosis in ADSCs in vitro

**Fig. 6:**
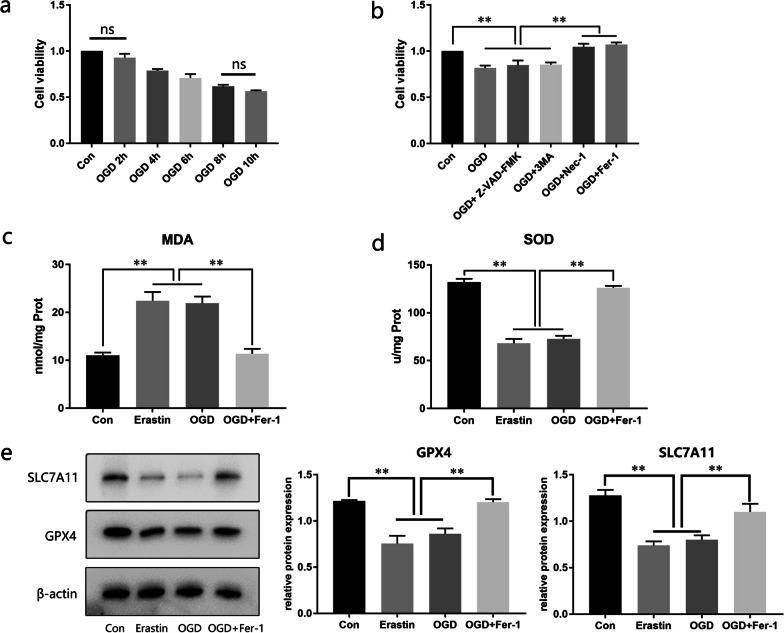
Effect of oxygen and glucose deprivation (OGD) culture on the levels of ferroptosis in adipose-derived stem cells (ADSCs). **a** Cell viability at 2, 4, 6, 8, and 10 h in the OGD model (n = 3). **b** Cell viability under OGD conditions with different inhibitors (n = 3). **c**, **d** Intracellular MDA and SOD levels in ADSCs (n = 3). (e, f) GPX4 and SLC7A11 expression in ADSCs (n = 3). Full-length blots are presented in Additional file 4. Data are presented as the mean ± SD. *p < 0.05, **p < 0.01

The cells employed in our experiment were identified as ADSCs by Flow cytometry and osteogenic and adipogenic induction (Additional file 3: Fig S2). With increasing OGD intervention time, the viability of ADSCs decreased (Fig. 6a). Based on the cell viability results, we selected 4 h as the OGD duration for subsequent experiments. We treated ADSCs with various inhibitors to determine the type of OGD-induced cell death. Notably, Fer-1 and Nec-1 prevented OGD-induced cell death, while inhibitor 3-MA and Z-VAD-FMK did not (Fig. 6b). Intracellular oxidative stress was enhanced in the OGD- and erastin-treated (positive control) group but was attenuated in the Fer-1 group, based on MDA and SOD levels (Fig. 6c, d). The western blot results indicated that OGD and erastin caused a decrease in SLC7A11 and GPX4 protein levels, which were rescued with the addition of Fer-1 (Fig. 6e).

## GSH reverses OGD-mediated ferroptosis by activating the SLC7A11/GPX4 axis

**Fig. 7:**
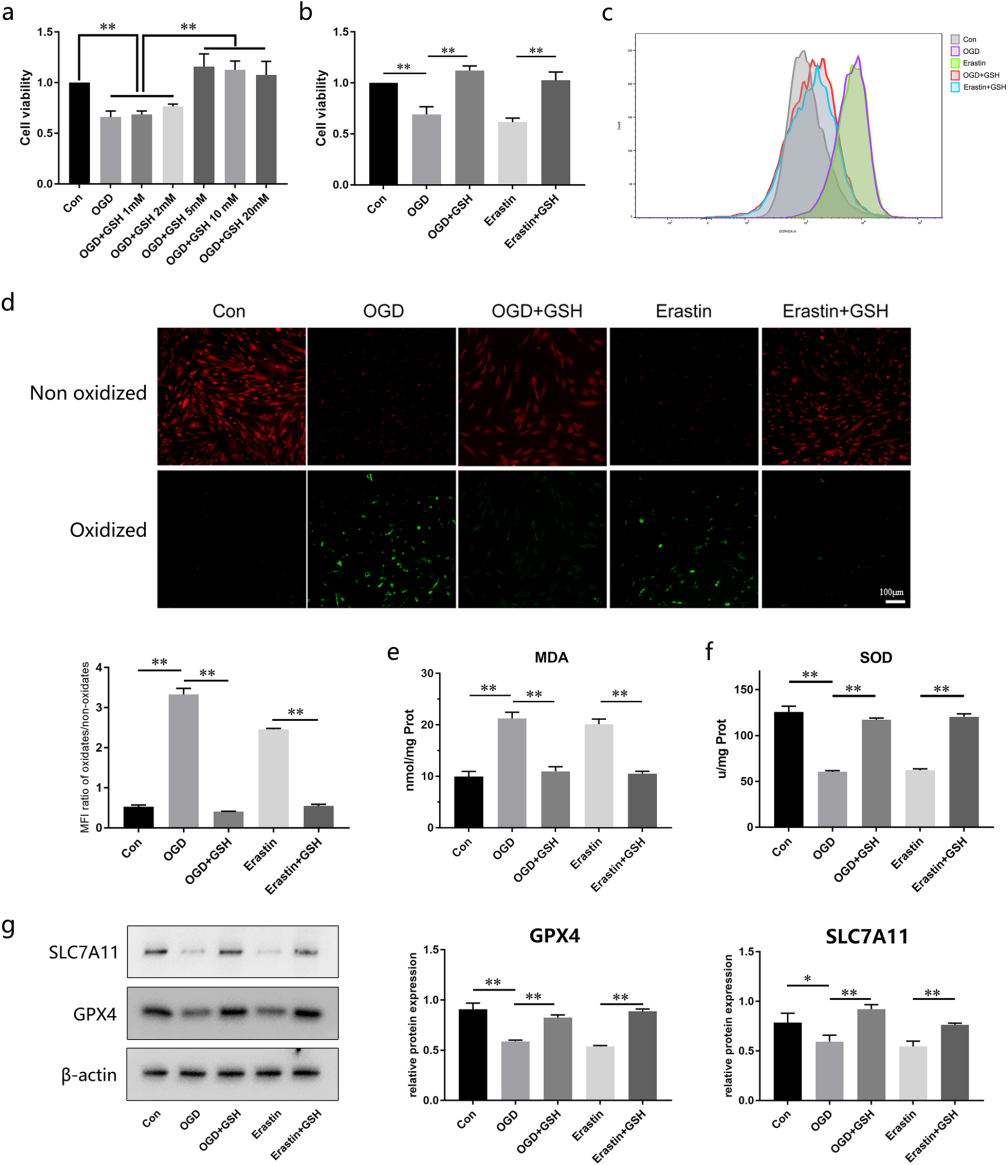
GSH rescues ADSCs from ferroptosis by activating the SLC7A11/GPX4 axis. **a** Cell viability in the OGD model at different concentrations of GSH (n = 3). **b** Attenuation of decreased cell viability in the OGD- and erastin-treated groups by GSH (n = 3). **c** Intracellular reactive oxygen species (ROS) levels determined by flow cytometry (n = 3). **d** BODIPY staining for lipid peroxidation in ADSCs (n = 3). **e**, **f** Intracellular MDA and SOD levels in each treatment group (n = 3). **g**, **h** Protein expression and quantification of GPX4, SLC7A11 by western blotting (n = 3). Full-length blots are presented in Additional file 4. Data are presented as the mean ± SD. *p < 0.05, **p < 0.01

We investigated the mechanism by which GSH enhances the resistance of ADSCs to ferroptosis. Pretreatment of cells with GSH significantly improved the viability of OGD-treated cells; 5 mM was confirmed to be the optimum GSH concentration (Fig. 7a). GSH also attenuated erastin-induced cell death, demonstrating its ferroptosis suppressing effects (Fig. 7b). Flow cytometry using the DCFH-DA probe indicated that the marked elevation in ROS levels caused by the OGD and erastin treatments was rescued by the GSH treatment (Fig. 7c). We visualized lipid peroxides (LPOs) in ADSCs by staining with a BODIPY 581/591 C11 probe and found that cells in the OGD- and erastin-treated groups showed extensive green staining, indicating the production of a large amount of LPOs, whereas GSH-supplemented cells displayed only sporadic green staining (Fig. 7d). GSH also reduced oxidative stress in ferroptotic ADSCs, based on MDA and SOD levels (Fig. 7e, f). The OGD and erastin treatments inhibited the activity of the antioxidant system by downregulating the activation of the SLC7A11/GPX4 axis. GSH abolished this inhibitory effect and promoted the recovery of SLC7A11 and GPX4 levels (Fig. 7g).

## Discussion

Researchers have long attempted to improve fat graft survival; however, no substantial progress has been made in clinical practice. In our opinion, this is mainly due to the fact that many laboratory-stage techniques are limited by safety and ethical concerns. Additionally, because the survival mechanism of fat grafts has not yet been fully elucidated, there is a lack of effective targeted interventions. In this study, we propose that the commonly applied antioxidant GSH can improve fat graft survival and confirmed that its protective effects were achieved by inhibition of ferroptosis.

Using MRI, we found that GSH maintained the cell retention, appearance, and tissue structure of fat grafts. MRI is an effective diagnostic tool that enables the characterization of a graft (e.g., whether it is solid or liquid; the extent and location of necrosis) without requiring an autopsy. It is also possible to describe the relationship between the graft and the host, i.e., whether the transplanted graft grows invasively or is locally confined [23]. T2-weighted imaging showed that the off-center region within the control grafts exhibited liquefaction-equivalent hyperintense signals with recognizable boundaries. This suggested that the superficial layer of the grafts had a greater viability compared to the deeper layers, consistent with Kotaro’s three zones theory [8]. In the periphery of the control grafts, we also observed hyperintense signals from small separate lobulated fat masses—structures that may eventually develop into cysts or foci of calcification and are a common clinical complication of fat grafting. However, these phenomena were not observed in the GSH-treated grafts, even in the early post-transplant period, confirming the potent and efficient protective effect of GSH. Although not commonly used in fat graft research, our findings indicated that MRI can be used for early graft prognosis, which can benefit early intervention and overall clinical management. It should be noted that both T1- and T2-weighted scans were performed in our study, but the T1 images were not clear enough for analysis.

We confirmed the occurrence of ferroptosis in the transplanted fat grafts, which was another important finding of our study. Ferroptosis—a more recently discovered type of programmed cell death—is distinct from apoptosis, necroptosis, or pyroptosis in terms of morphology, biochemistry, and development. It is driven by the accumulation of iron-dependent LPOs. Ferroptosis is characterized by the dysregulation of oxidant and antioxidant levels, which leads to plasma membrane damage. Shrunken mitochondria with condensed ruptured outer membranes as well as vestigial cristae are a physical hallmark of ferroptosis [24]. Moreover, ferroptosis is increasingly recognized as an important driver of disease pathogenesis, including but not limited to cancer, degenerative diseases, ischemic injury, and infections [25]. However, the role of ferroptosis in fat graft survival was hitherto unclear. In this study, we confirmed our hypothesis that ferroptosis was involved in the survival of fat grafts. First, RNA-seq confirmed that the DEGs were mainly enriched in the ferroptosis pathway. Subsequent experiments indicated that control grafts showed features associated with exacerbated ferroptosis, including elevated LPO levels and suppression of the antioxidant system. In addition to the effects of necrosis and apoptosis, other regulatory pathways, such as autophagy and necroptosis, have been proposed to play important roles in fat graft survival [26, 27]. Although we did not investigate each of these cell death pathways, we do not deny their potential roles. Future studies should consider the different forms of cell death, whether they act simultaneously or successively through interactions, and whether any one of these cell death pathways plays a predominant role.

In this study, GSH inhibited ferroptosis by activating the SLC7A11/GPX4 axis and reducing LPO levels to ultimately alleviate graft injury and promote survival. This was also confirmed in vitro, where GSH promoted the resistance of ADSCs to ferroptosis. SLC7A11 is a key component of the cystine/glutamate antiporter system Xc–, which imports cystine for GSH biosynthesis. In the presence of GSH, GPX4 mediates the conversion of toxic LPOs to nontoxic lipid alcohols. SLC7A11 inhibition results in GSH depletion, which downregulates GPX4 levels, thereby promoting cellular/subcellular membrane damage caused by LPOs [28]. The SLC7A11/GPX4 axis, serving as a pivotal inhibitory pathway in the orchestration of ferroptosis, has garnered substantial attention. It is increasingly recognized as a promising therapeutic target for diseases linked to ferroptosis, and its potential to enhance outcomes of fat transplantation has come to light [29, 30]. Our study provides strong evidences, elucidating that the induction of ferroptosis resistance as an effective strategy to promote the survival of transplanted fat grafts.

It was demonstrated in in vitro experiments that OGD effectively induced the death of ADSCs. To gain deeper insights into the precise classification of cell death, we employed a diverse array of inhibitors. Caspases assume a pivotal role in apoptosis, serving as primary executors through the cleavage of cellular proteins, thereby instigating and executing the apoptotic cascade. Hence, we employed the broad spectrum inhibitor Z-VAD-FMK to selectively target caspases and impede the apoptotic pathway. 3-MA stands as a commonly employed autophagy inhibitor. Through the inhibition of phosphoinositide 3-kinase (PI3K), 3-MA disrupts the formation of autophagosomes, thereby impeding the initiation of the autophagic process. Nec-1 functions through the specifically targeting of Receptor-Interacting Protein Kinase 1 (RIPK1), inducing disruption in the assembly of the necrosome, consequently impeding the execution of necroptotic cell death. Fer-1 operates as a ferroptosis inhibitor, predominantly through the scavenging of lipid peroxides. In this capacity, it serves to shield cells from oxidative harm, thereby contributing to the preservation of cellular membrane integrity. Within these comparatively autonomous mechanisms governing cell death, the introduction of Fer-1 substantially elevated cell survival rates, indicative of the effective suppression of OGD induced ferroptosis. Additionally, we noted the inhibition of cell death with Nec-1, suggesting that OGD has the potential to induce necroptosis, aligning with findings by Qi et al. [27]. Thus, our study provides additional substantiation for the appropriateness of this model in simulating the environment wherein the replanted grafts reside post-transplantation, and investigating the mechanisms governing cell death.

Since the conception of cell-assisted lipotransfer by Yoshimura in 2006 [31], numerous approaches have been proposed to improve the long-term efficacy of lipotransfers. Based on our results, we propose that GSH-assisted lipotransfer could be a safe and effective alternative to traditional procedures. GSH has long been used in clinical practice via various routes of administration, with good therapeutic effects and safety [32–34]. Therefore, it can be applied directly as a topical (graft washing), oral supplement, subcutaneous/intravenous injection, or a combination of multiple routes (similar to the approach applied in our study). However, the optimization of treatment route and dosage requires further research. A previous study showed that graft washing induced better survival [35], which, if confirmed, could reduce the side effects related to local injections.

## Conclusions

This study describes a novel lipotransfer approach and our findings complemented the existing fat graft survival theory. GSH is a promising and clinically feasible drug that promotes fat graft survival by inhibiting ferroptosis via the SLC7A11/GPX4 axis. This study is the first to describe the role of ferroptosis in fat graft survival and demonstrate the potential of this pathway as a therapeutic target for improving lipotransfer success. Further research and clinical studies are needed to evaluate the performance of this GSH-based modification in fat transplantation.

### Supplementary Information

**Additional file1**: Primers used for PCR

**Additional file2**: KEGG pathway results related to “cell growth and death” and “aging.” The ferroptosis pathway ranked first

**Additional file3**: Identification of ADSCs. (a) Analysis of ADSCs surface markers by flow cytometry on positive markers of CD73, CD90, CD105, and negative markers CD11b, CD19, CD34, CD45, HLA-DR. (b) ADSCs were indentified for adipogenic differentiation by oil red O. (c) Osteogenic differentiation stained with Alizarin Red.

**Additional file4**: Full-length gels and blots

## Data Availability

The RNA-sequencing data has been uploaded to NCBI's Gene Expression Omnibus (GEO) and can be accessed by public (https://www.ncbi.nlm.nih.gov/geo/query/acc.cgi?acc=GSE231511). All data generated or analyzed in this study are included in this article.

## Abbreviations

3-MA: 3-Methyladenine
ADSC: Adipose-derived stem cell
DEG: Differentially expressed gene
ROS: Reactive oxygen species
LPOs: Lipid peroxides
OGD: Oxygen and glucose deprivation
MDA: Malondialdehyde
SOD: Superoxide dismutase
Fer-1: Ferrostatin-1
GSH: Glutathione
H&E: Hematoxylin and eosin
KEGG: Kyoto Encyclopedia of Genes and Genomes
MRI: Magnetic resonance imaging
NAC: *N*-Acetylcysteine
Nec-1: Necrostatin-1
NS: Normal saline
PBS: Phosphate-buffered saline
qRT-PCR: Quantitative real-time polymerase chain reaction
SD: Standard deviation
TEM: Transmission electron microscopy

## References

[1] Smith P, Adams WP, Lipschitz AH, Chau B, Sorokin E, Rohrich RJ, Brown SA (2006). Autologous human fat grafting: effect of harvesting and preparation techniques on adipocyte graft survival. Plast Reconstr Surg.

[2] Zhu M, Zhu M, Wu X, Xu M, Fan K, Wang J, Zhang L, Yin M, Wu J, Zhu Z, Yang G (2021). Porcine acellular dermal matrix increases fat survival rate after fat grafting in nude mice. Aesthetic Plast Surg.

[3] Franco R, Cidlowski JA (2012). Glutathione efflux and cell death. Antioxid Redox Signal.

[4] Saitoh T, Satoh H, Nobuhara M, Machii M, Tanaka T, Ohtani H, Saotome M, Urushida T, Katoh H, Hayashi H (2011). Intravenous glutathione prevents renal oxidative stress after coronary angiography more effectively than oral N-acetylcysteine. Heart Vessels.

[5] Tanzilli G, Arrivi A, Placanica A, Viceconte N, Cammisotto V, Nocella C, Barillà F, Torromeo C, Pucci G, Acconcia MC, Granatelli A (2021). Glutathione infusion before and 3 days after primary angioplasty blunts ongoing NOX2-mediated inflammatory response. J Am Heart Assoc.

[6] Søndergård SD, Cintin I, Kuhlman AB, Morville TH, Bergmann ML, Kjær LK, Poulsen HE, Giustarini D, Rossi R, Dela F, Helge JW (2021). The effects of 3 weeks of oral glutathione supplementation on whole body insulin sensitivity in obese males with and without type 2 diabetes: a randomized trial. Appl Physiol Nutr Metab.

[7] Miyachi T, Tsuchiya T, Oyama A, Tsuchiya T, Abe N, Sato A, Chiba Y, Kurihara S, Shibakusa T, Mikami T (2013). Perioperative oral administration of cystine and theanine enhances recovery after distal gastrectomy: a prospective randomized trial. J Parenter Enteral Nutr.

[8] Eto H, Kato H, Suga H, Aoi N, Doi K, Kuno S, Yoshimura K (2012). The fate of adipocytes after nonvascularized fat grafting: evidence of early death and replacement of adipocytes. Plast Reconstr Surg.

[9] Gillis J, Gebremeskel S, Phipps KD, MacNeil LA, Sinal CJ, Johnston B, Hong P, Bezuhly M (2015). Effect of N-acetylcysteine on adipose-derived stem cell and autologous fat graft survival in a mouse model. Plast Reconstr Surg.

[10] Pietruski P, Paskal W, Paluch Ł, Paskal AM, Nitek Ż, Włodarski P, Walecki J, Noszczyk B (2021). The impact of n-acetylcysteine on autologous fat graft: first-in-human pilot study. Aesthetic Plast Surg.

[11] Hao X, Zhang T, Yang Y, Feng H, Wang Y, Song Y, Su Y, Guo S (2015). Morphological features of cell death and tissue remolding of fat grafts. Ann Plast Surg.

[12] Herold C, Rennekampff HO, Engeli S (2013). Apoptotic pathways in adipose tissue. Apoptosis.

[13] Witort EJ, Pattarino J, Papucci L, Schiavone N, Donnini M, Lapucci A, Lulli M, Russo GL, Mori A, Dini M, Capaccioli S (2007). Autologous lipofilling: coenzyme Q10 can rescue adipocytes from stress-induced apoptotic death. Plast Reconstr Surg.

[14] Kim J, Park M, Jeong W, Lee HW, Lee G, Lee KS, Park SW, Choi J (2019). Recipient-site preconditioning with deferoxamine increases fat graft survival by inducing VEGF and neovascularization in a rat model. Plast Reconstr Surg.

[15] Kitakata H, Endo J, Matsushima H, Yamamoto S, Ikura H, Hirai A, Koh S, Ichihara G, Hiraide T, Moriyama H, Shirakawa K (2021). MITOL/MARCH5 determines the susceptibility of cardiomyocytes to doxorubicin-induced ferroptosis by regulating GSH homeostasis. J Mol Cell Cardiol.

[16] Kennedy L, Sandhu JK, Harper ME, Cuperlovic-Culf M (2020). Role of glutathione in cancer: from mechanisms to therapies. Biomolecules.

[17] Niu B, Liao K, Zhou Y, Wen T, Quan G, Pan X, Wu C (2021). Application of glutathione depletion in cancer therapy: enhanced ROS-based therapy, ferroptosis, and chemotherapy. Biomaterials.

[18] Stockwell BR, Angeli JPF, Bayir H, Bush AI, Conrad M, Dixon SJ, Fulda S, Gascón S, Hatzios SK, Kagan VE, Noel K (2017). Ferroptosis: a regulated cell death nexus linking metabolism, redox biology, and disease. Cell.

[19] Mou S, Zhou M, Li Y, Wang J, Yuan Q, Xiao P, Sun J, Wang Z (2019). Extracellular vesicles from human adipose-derived stem cells for the improvement of angiogenesis and fat-grafting application. Plast Reconstr Surg.

[20] Shoshani O, Livne E, Armoni M, Shupak A, Berger J, Ramon Y, Fodor L, Gilhar A, Peled IJ, Ullmann Y (2005). The effect of interleukin-8 on the viability of injected adipose tissue in nude mice. Plast Reconstr Surg.

[21] Li Z, Li S, Li K, Jiang X, Zhang J, Liu H (2020). A highly simulated scar model developed by grafting human thin split-thickness skin on back of nude mouse: the remodeling process, histological characteristics of scars. Biochem Biophys Res Commun.

[22] Li Z, Zhang J, Li M, Tang L, Liu H (2020). Concentrated nanofat: A modified fat extraction promotes hair growth in mice via the stem cells and extracellular matrix components interaction. Ann Transl Med.

[23] Chan LP, Gee R, Keogh C, Munk PL (2003). Imaging features of fat necrosis. AJR Am J Roentgenol.

[24] Chen X, Kang R, Kroemer G, Tang D (2021). Organelle-specific regulation of ferroptosis. Cell Death Differ.

[25] Li D, Li Y (2020). The interaction between ferroptosis and lipid metabolism in cancer. Signal Transduct Target Ther.

[26] Pang H, Zhou Y, Wang J, Wu H, Liu X, Gao F, Xiao Z (2021). Berberine influences the survival of fat grafting by inhibiting autophagy and apoptosis of human adipose derived mesenchymal stem cells. Drug Des Dev Ther.

[27] Yang Z, Qi Z, Yang X, Gao Q, Hu Y, Yuan X (2022). Inhibition of RIP3 increased ADSC viability under OGD and modified the competency of adipogenesis, angiogenesis, and inflammation regulation. Biosci Rep.

[28] Chen X, Yu C, Kang R, Kroemer G, Tang D (2021). Cellular degradation systems in ferroptosis. Cell Death Differ.

[29] Pan Y, Wang X, Liu X, Shen L, Chen Q, Shu Q (2022). Targeting ferroptosis as a promising therapeutic strategy for ischemia-reperfusion injury. Antioxidants (Basel).

[30] Liu M, Kong XY, Yao Y, Wang XA, Yang W, Wu H, Li S, Ding JW, Yang J (2022). The critical role and molecular mechanisms of ferroptosis in antioxidant systems: a narrative review. Ann Transl Med.

[31] Matsumoto D, Sato K, Gonda K, Takaki Y, Shigeura T, Sato T, Aiba-Kojima E, Iizuka F, Inoue K, Suga H, Yoshimura K (2006). Cell-assisted lipotransfer: supportive use of human adipose-derived cells for soft tissue augmentation with lipoinjection. Tissue Eng.

[32] Sonthalia S, Daulatabad D, Sarkar R (2016). Glutathione as a skin whitening agent: facts, myths, evidence and controversies. Indian J Dermatol Venereol Leprol.

[33] Wu JH, Batist G (2013). Glutathione and glutathione analogues; therapeutic potentials. Biochim Biophys Acta.

[34] Bump EA, Al-Sarraf R, Pierce SM, Coleman CN (1992). Elevation of mouse kidney thiol content following administration of glutathione. Radiother Oncol.

[35] Chen Y, Chai Y, Yin B, Zhang X, Han X, Cai L, Yin N, Li F (2022). Washing lipoaspirate improves fat graft survival in nude mice. Aesthetic Plast Surg.

