# Supplementary material for: Glutathione supplementation improves fat graft survival by inhibiting ferroptosis via the SLC7A11/GPX4 axis
Source: Stem Cell Res Ther. 2024 Jan 30;15:25. doi: 10.1186/s13287-024-03644-0 (PMC10826280; doi:10.1186/s13287-024-03644-0)
Supplement: Supplementary file 1 — Additional file1: Primers used for PCR [file 13287_2024_3644_MOESM1_ESM.docx]

| GAPDH | Forward | CCTCGTCCCGTAGACAAAATG | 133 bp |
| --- | --- | --- | --- |
|  | Reverse | TGAGGTCAATGAAGGGGTCGT |  |
| Fth1 | Forward | GCCAAATACTTTCTCCACCAATC | 244 bp |
|  | Reverse | TGAAGTCACATAAGTGGGGATCATT |  |
| Gclc | Forward | CATCCTCCAGTTCCTGCACATC | 143 bp |
|  | Reverse | CATCGCCTCCATTCAGTAACAAC |  |
| Gclm | Forward | TTCGCCTCCGATTGAAGATG | 177 bp |
|  | Reverse | TGGTTACTATTGGGTTTTACCTGTG |  |
| Gss | Forward | TATTTGACCAGCGTGCCGTAG | 298 bp |
|  | Reverse | AGACCCACCCTGCTCAGTTC |  |
| Hmox1 | Forward | AACTAGCCCAGTCCGGTGATG | 151 bp |
|  | Reverse | CTCTGGACACCTGACCCTTCTG |  |
| Slc7a11 | Forward | GCTATCATCACAGTGGGCTACG | 206 bp |
|  | Reverse | TAGAATAACCTGGAGACAGCGAAC |  |
| Slc39a14 | Forward | ATTGCCCTAGCCGATATGTTCC | 132bp |
|  | Reverse | GAGGACCAGCATAATGGAGAAGC |  |
| Slc40a1 | Forward | TCACCTAAAGATACTGAGCCAAAAC | 142 bp |
|  | Reverse | CATCTCGGAAAGTGCGGAAG |  |

Table 1. Primers used for PCR
