# Supplementary figures and images for: Glutathione supplementation improves fat graft survival by inhibiting ferroptosis via the SLC7A11/GPX4 axis
Source: Stem Cell Res Ther. 2024 Jan 30;15:25. doi: 10.1186/s13287-024-03644-0 (PMC10826280; doi:10.1186/s13287-024-03644-0)

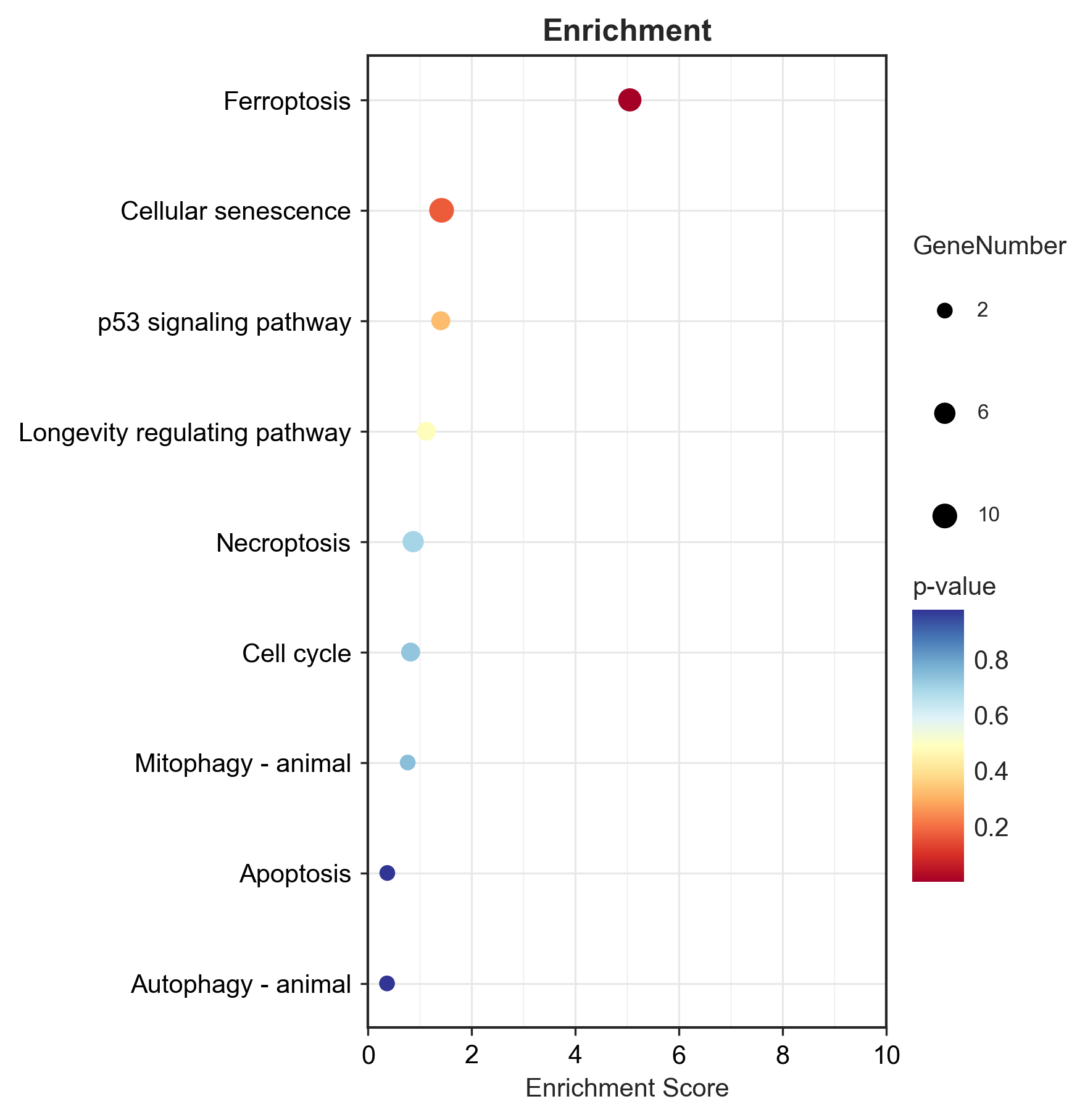

Supplement: Supplementary file 2 — Additional file2: KEGG pathway results related to “cell growth and death” and “aging.” The ferroptosis pathway ranked first [file 13287_2024_3644_MOESM2_ESM.tif]

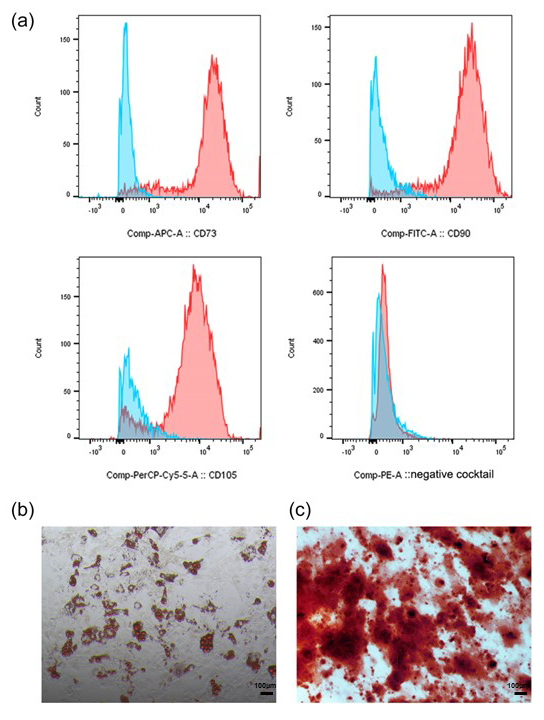

Supplement: Supplementary file 3 — Additional file3: Identification of ADSCs. (a) Analysis of ADSCs surface markers by flow cytometry on positive markers of CD73, CD90, CD105, and negative markers CD11b, CD19, CD34, CD45, HLA-DR. (b) ADSCs were indentified for adipogenic differentiation by oil red O. (c) Osteogenic differentiation stained with Alizarin Red. [file 13287_2024_3644_MOESM3_ESM.jpg]
