# Supplementary material for: Glutathione supplementation improves fat graft survival by inhibiting ferroptosis via the SLC7A11/GPX4 axis
Source: Stem Cell Res Ther. 2024 Jan 30;15:25. doi: 10.1186/s13287-024-03644-0 (PMC10826280; doi:10.1186/s13287-024-03644-0)

**Fig5 gels and blots**

PPARγ


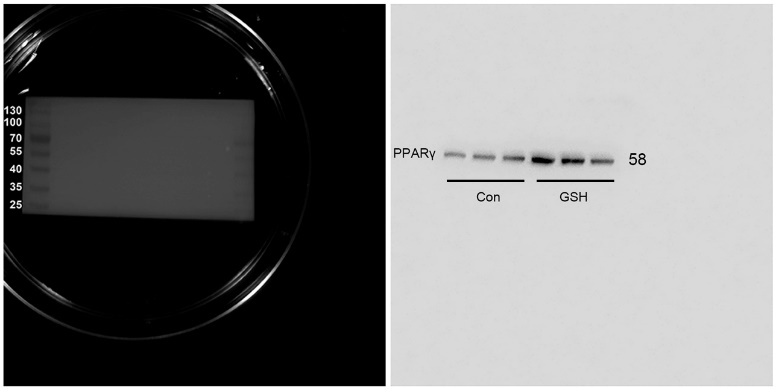


SLC7A11


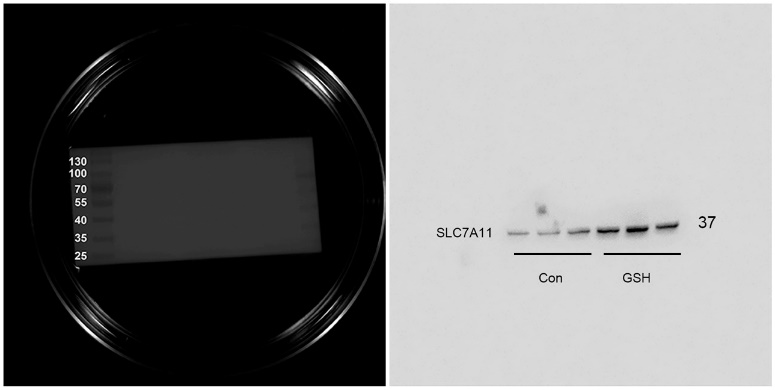


GPX4


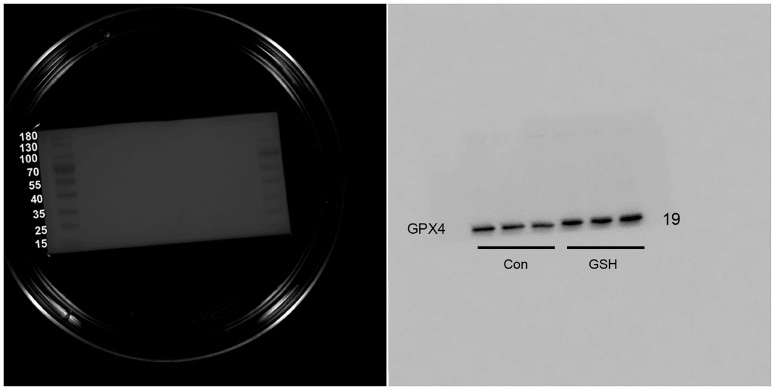


β-actin


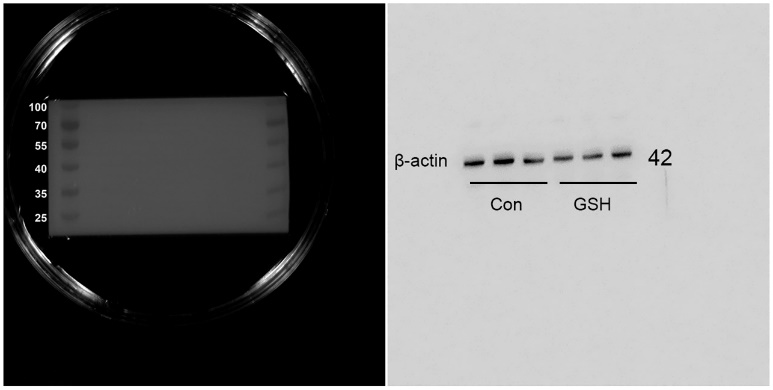


**Fig6 gels and blots**

SLC7A11


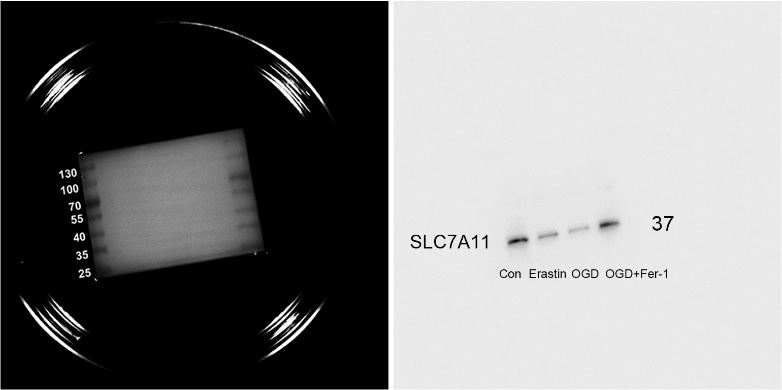


GPX4


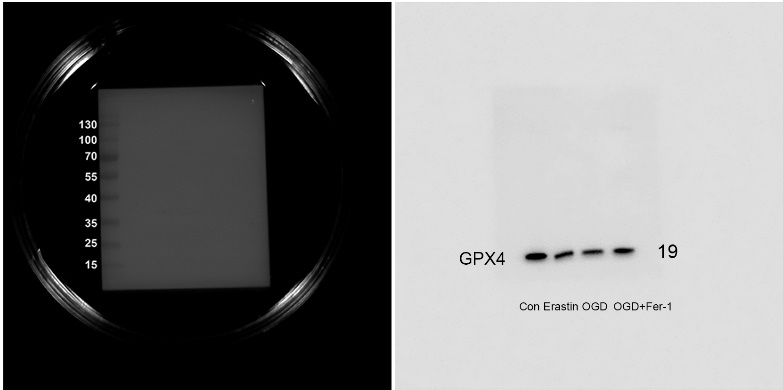


β-actin


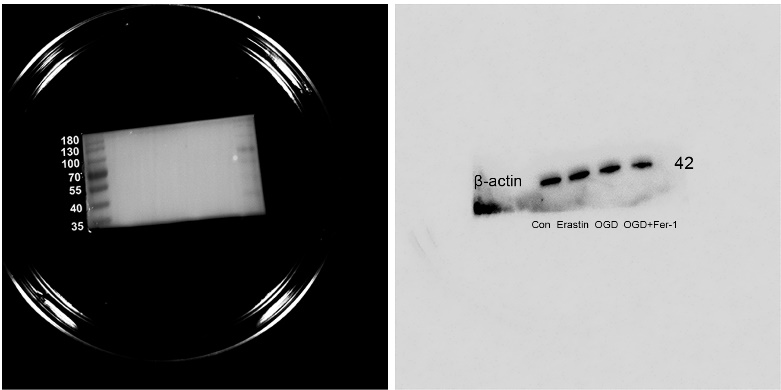


**Fig7 gels and blots**

SLC7A11


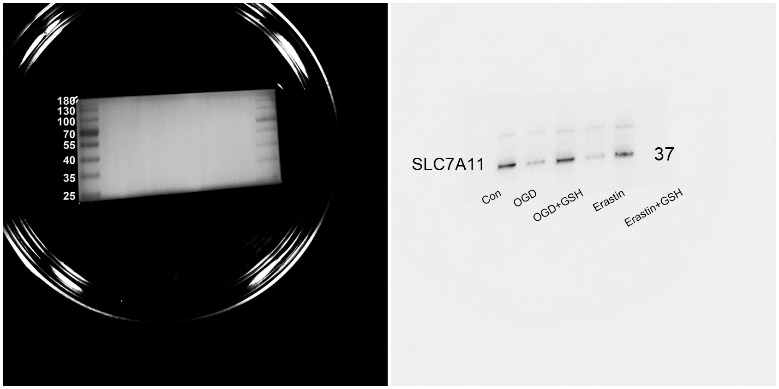


GPX4


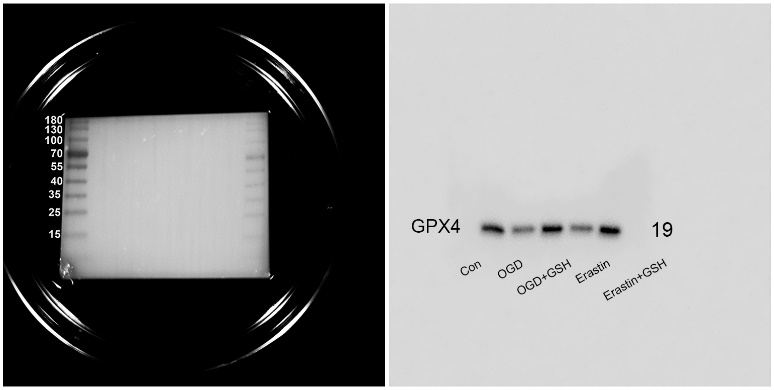


β-actin


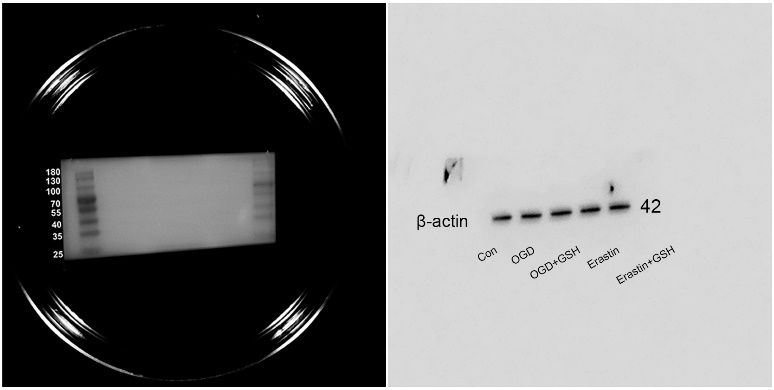

Supplement: Supplementary file 4 — Additional file4: Full-length gels and blots [file 13287_2024_3644_MOESM4_ESM.docx]
